# Small-bowel lymphangioma with mesenteric cystic extension causing acute obstruction in an adult: a case report

**DOI:** 10.1093/jscr/rjag987

**Published:** 2026-10-30

**Authors:** Gopi Gopalapillai, Khoula Al-Harrasi

**Affiliations:** General Surgery, Royal Brisbane and Women's Hospital, Metro North Health, Herston, QLD 4029, Australia; General Surgery, Royal Brisbane and Women's Hospital, Metro North Health, Herston, QLD 4029, Australia

**Keywords:** lymphangioma, lymphatic malformation, small-bowel obstruction, mesenteric cyst, volvulus

## Abstract

Small-bowel lymphangioma is rare in adults and only exceptionally presents with acute obstruction. A 45-year-old man with no previous abdominal surgery presented with sudden severe abdominal pain, nausea, and vomiting. Computed tomography demonstrated a fluid-density pelvic lesion with a single transition point, twisting of adjacent small bowel and mesentery, proximal dilatation, and mesenteric congestion. Emergency laparoscopy identified bowel twisted around a mesenteric cyst and was converted to laparotomy because distension prevented safe reduction. The lesion was inseparable from the mesentery supplying a 20-cm segment, which was resected en bloc. Histology demonstrated D2-40-positive lymphatic channels extending from mucosa to subserosa and communicating with the mesenteric cyst, confirming lymphangioma. Recovery was uncomplicated and the patient was discharged on postoperative day 7. This case highlights lymphatic malformation as a rare pathological lead point for obstruction in a virgin abdomen.

## Introduction

Small intestinal lymphangioma, now generally classified as a lymphatic malformation, is rare in adults. A recent review of 97 adult cases found abdominal pain and gastrointestinal bleeding to be the dominant presentations, while acute mechanical obstruction was much less common [1]. Reported obstructive mechanisms include torsion, volvulus, closed-loop obstruction, and intussusception [2–4]. We report a 45-year-old man with a virgin abdomen in whom lymphangioma involved the small-bowel wall and communicated with a mesenteric cyst that acted as the lead point for acute obstruction.

## Case report

On 24 May 2025, a 45-year-old man with no previous abdominal surgery presented with sudden, progressively worsening periumbilical pain, nausea, and three episodes of vomiting. He was afebrile and haemodynamically stable. The abdomen was soft with marked epigastric and periumbilical tenderness and voluntary guarding, without rebound or percussion tenderness. White-cell count was 14.2 × 10^9^/l (neutrophils 11.92 × 10^9^/l); haemoglobin, platelets, renal and liver function, electrolytes, and lipase were normal.

Computed tomography (CT) showed a well-demarcated lobulated fluid-density pelvic lesion extending into the right iliac fossa (Fig. 1). The stomach and small bowel were dilated, with a single right lower-quadrant transition point at the lesion’s superomedial margin. Bowel and mesenteric fat were twisted around the lesion with mesenteric congestion; distal small bowel and colon were collapsed. There was no abnormal bowel-wall enhancement, pneumatosis, or portal venous gas. The radiological differential included mesenteric cyst, peritoneal inclusion cyst, and lymphocele.

**Figure 1:**
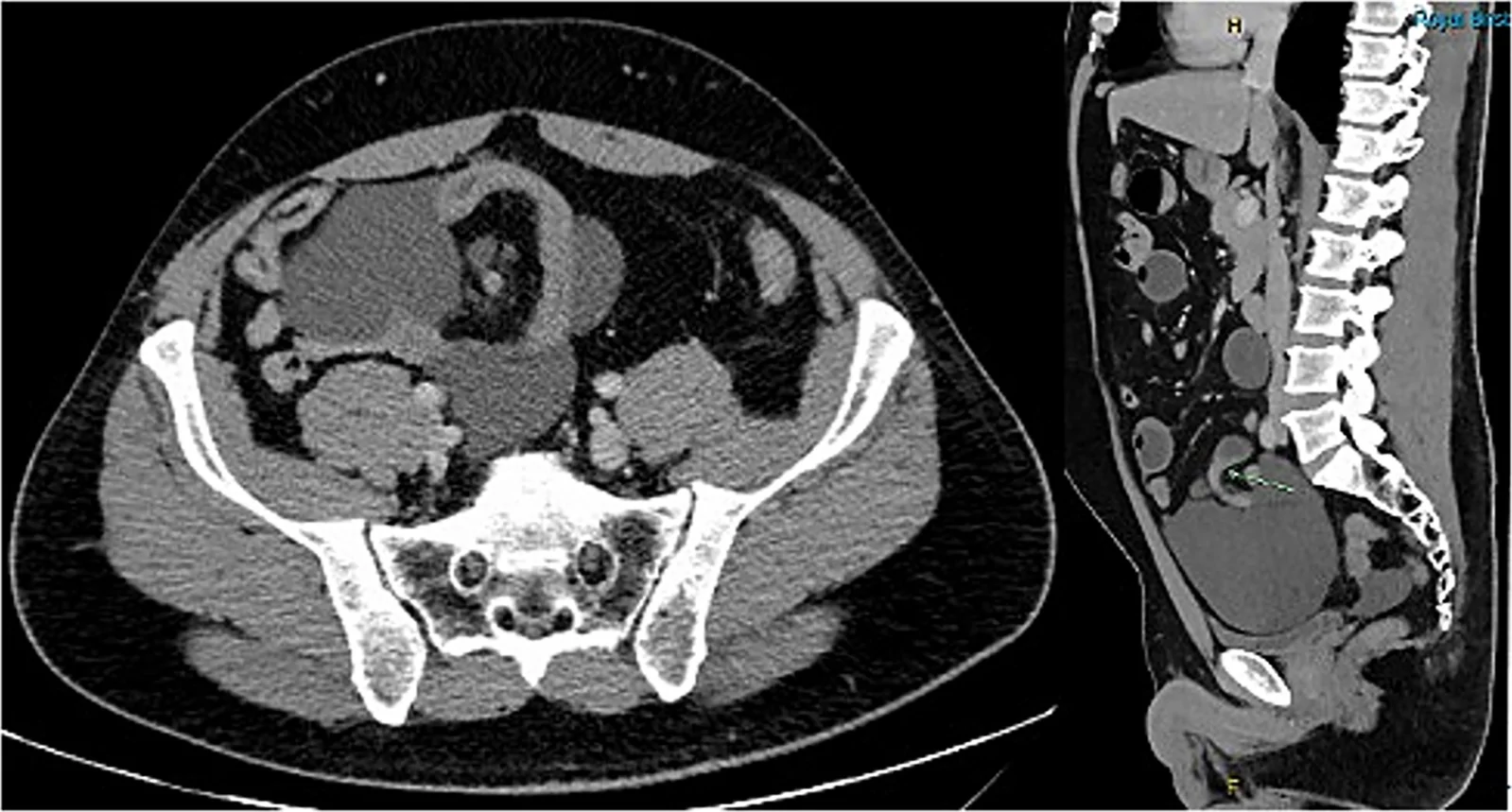
Preoperative portal venous phase CT. (a) Axial image demonstrates a well-demarcated fluid-density pelvic lesion extending into the right iliac fossa. (b) Sagittal image demonstrates the cystic lesion at the small-bowel transition point with adjacent bowel and mesenteric twisting and proximal dilatation (arrow).

Emergency laparoscopy on 25 May 2025 identified clear serous fluid and a large mesenteric cyst around which small bowel had twisted, with proximal dilatation and distal collapse (Fig. 2). Marked distension prevented safe reduction and assessment, prompting conversion to midline laparotomy. The lesion lay approximately 100 cm from the duodenojejunal flexure and involved the mesentery supplying a 20-cm bowel segment. As it could not be separated without compromising perfusion, the segment and cyst were resected en bloc with a stapled side-to-side anastomosis.

**Figure 2:**
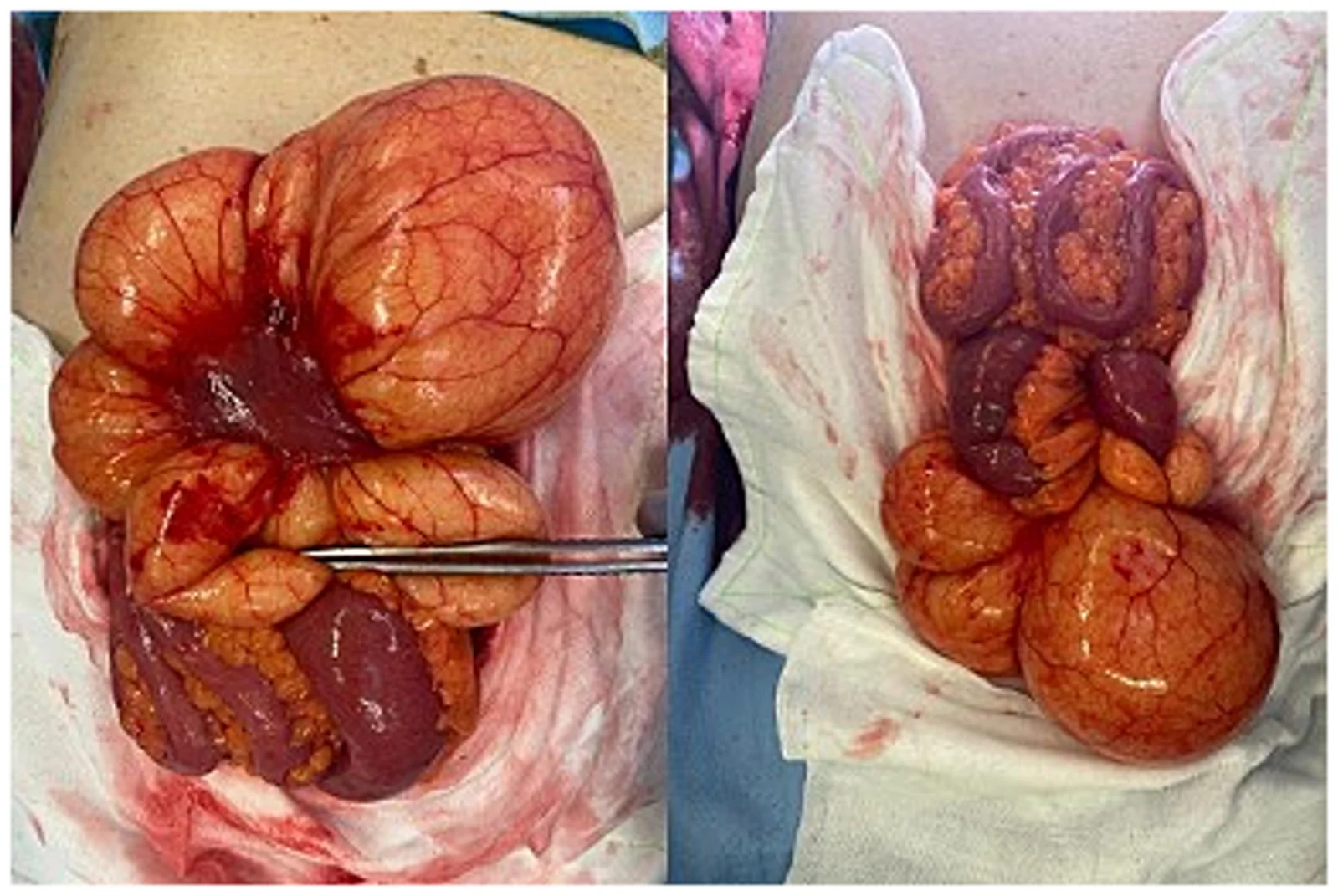
Intraoperative macroscopic appearance of the involved small bowel and mesenteric cystic lesion. Two views demonstrate the large smooth cystic mesenteric component associated with the affected small-bowel segment before en bloc resection.

Macroscopy demonstrated a 190-mm small-bowel segment with a 75 × 60 × 10-mm collapsed mesenteric cyst and adjacent 5–10-mm pale mucosal polyps. Histology showed variably dilated lymphatic channels extending from the lamina propria through the full thickness of the bowel wall to the subserosa and communicating with the large unilocular mesenteric cyst (Fig. 3a). A higher-power H&E image demonstrated ectatic lymphatic channels within the superficial lamina propria, lined by bland flattened endothelial cells (Fig. 3b). The channels were D2-40 positive and endothelial nuclei were highlighted by ERG; no malignancy was identified and excision was complete. Peritoneal fluid cytology was negative for malignant cells. Recovery was uncomplicated: flatus passed from postoperative day 1, the nasogastric tube was removed on day 2 and the drain on day 5. He was discharged on postoperative day 7 and subsequently discharged from surgical follow-up after review of the benign, completely excised pathology.

**Figure 3:**
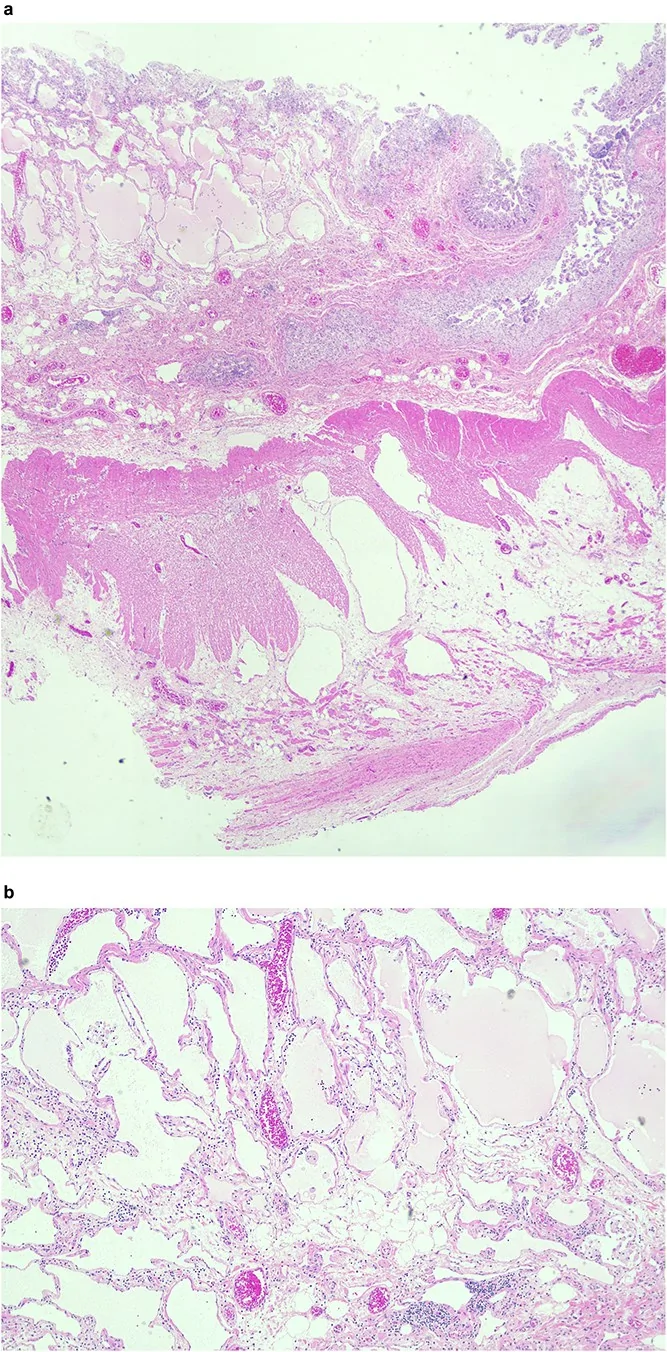
Histopathology of small-bowel lymphangioma. (a) Low-power stitched composite haematoxylin and eosin (H&E) image demonstrating full-thickness involvement of the small bowel by variably dilated lymphatic channels; the deep aspect shows the wall and lining of the large mesenteric cystic component. A single magnification is not applicable because this is a composite image. (b) Higher-power H&E image (40×) of the superficial component showing ectatic lymphatic channels within the lamina propria, lined by bland flattened endothelial cells.

## Discussion

Adult small-bowel lymphatic malformations are uncommon and heterogeneous in site and morphology. In the recent 97-case review, lesions were most often jejunal or mesenteric, CT detected a lesion in 87%, and segmental bowel resection was the most frequent treatment [1]. The pathological architecture is central to this case: low-power H&E demonstrates lymphatic proliferation through the full thickness of the bowel wall with continuity to the mesenteric cystic component, distinguishing it from an isolated mesenteric cyst (Fig. 3a). The superficial lamina propria proliferation seen at higher power (Fig. 3b) also explains the adjacent pale mucosal polyps seen macroscopically.

The distinguishing feature of this case is its mechanism of presentation. A 2024 *Journal of Surgical Case Reports* publication described distal-ileal lymphangioma in a 49-year-old woman with abdominal pain but preserved bowel function and no vomiting; imaging suggested a mesenteric lymphangioma before resection, and histology showed lymphatic spaces in the bowel wall and adjacent mesenteric fat [5]. More recently, Singh *et al*. reported small-bowel obstruction from cystic mesenteric lymphangioma in a man with a virgin abdomen but longstanding gastrointestinal symptoms [6]. Our patient instead presented with abrupt acute obstruction, CT demonstrated a single transition point with bowel and mesenteric fat twisting around the cyst, and histology showed channels spanning mucosa to subserosa with direct communication to the mesenteric cyst. This combination defines the teaching value of the present report without implying that mesenteric lymphangioma itself is novel.

Preoperative classification of an abdominal cystic lesion is difficult because lymphatic malformations overlap with mesenteric cysts, lymphoceles, and other benign cystic processes. Typical CT appearances are low-attenuation, thin-walled uni- or multiloculated lesions, but these findings are not specific [7, 8]. In an obstructed patient, the clinically decisive feature may therefore be the relationship of the lesion to the transition point. Here, twisting of bowel and mesenteric fat around the cyst, complete distal collapse, and mesenteric congestion identified a pathological mechanical lead point. Although bowel-wall enhancement remained preserved and there was no pneumatosis or portal venous gas, the configuration raised concern for progression to vascular compromise and justified urgent exploration.

Operative strategy is determined by bowel involvement and the mesenteric vascular arcade. Enucleation may be possible when a cystic lesion separates safely from adjacent vessels, whereas segmental resection is required when the lesion involves the bowel wall or its supplying mesentery [2–6]. Here, laparoscopy established the mechanical cause but marked distension prevented safe reduction and full assessment; conversion to laparotomy allowed controlled untwisting, inspection, and en bloc resection with viable margins. The histopathological combination of bland, dilated channels, D2-40 positivity, and ERG-positive endothelial nuclei confirmed lymphatic differentiation [5, 8–10].

Complete excision is important because recurrence is predominantly associated with residual or diffuse disease. In the 2026 review, recurrence occurred in 11% overall but was not reported after complete excision [1]. There is no established surveillance schedule after complete resection of a localized benign lesion. For surgeons, the practical message is that a virgin abdomen does not exclude a mechanical lead point: when a cystic mesenteric lesion is anatomically linked to a transition point and twisting, prompt exploration may both relieve the obstruction and establish the diagnosis.

## Data Availability

All relevant de-identified clinical information is contained within the article. Additional patient-level data are not publicly available to protect patient privacy.
